# Assessment of Sick Building Syndrome and Its Associating Factors Among Nurses in the Educational Hospitals of Shahid Sadoughi University of Medical Sciences, Yazd, Iran

**DOI:** 10.5539/gjhs.v7n2p247

**Published:** 2014-11-16

**Authors:** Mohammad Reza Vafaeenasab, Mohammad Ali Morowatisharifabad, Mohammad Taghi Ghaneian, Mahdi Hajhosseini, Mohammad Hassan Ehrampoush

**Affiliations:** 1 Department of Physical and Rehabilitation Medicine, Shahid Sadoughi University of Medical Sciences, Yazd, Iran; 2 Department of Health Education & Promotion, Shahid Sadoughi University of Medical Sciences, Yazd, Iran; 3 Department of Environmental Health, Shahid Sadoughi University of Medical Sciences, Yazd, Iran; 4 International Campus, Shahid Sadoughi University of Medical Sciences, Yazd, Iran; 5 Department of Environmental Health, Shahid Sadoughi University of Medical Sciences, Yazd, Iran

**Keywords:** sick building syndrome, nurse, hospital, Yazd

## Abstract

**Background:**

Sick Building Syndrome is a diseases associated with indoor air quality accompanied with symptoms such as headache, dizziness, nausea, coughing and sneezing, irritation of eyes, throat and nose mucous membrane, and skin itching and inflammation. The purpose of this study was to investigate the symptoms of the syndrome and its related factors among nurses in teaching hospitals of Shahid Sadoughi University of Medical Sciences, Yazd, Iran.

**Methods:**

The study was conducted amongst nurses of three teaching hospitals of Shahid Sadoughi University of Medical Sciences, in Yazd. In this study the MM040EA questionnaire of sick building syndrome and indoor air quality was used and data extracted from the questionnaires were analyzed using the chi-square test and t-test.

**Results:**

The prevalence of sick building syndrome was 86.4%. The prevalence of the syndrome was in no association with age, gender, employment history and type of shift work. The most common symptoms reported by nurses included headache, fatigue and dry hands. Lack of sense of airflow, unpleasant odor in workplace (P<0.05), and also the amount of workload (P<0.001) were in relation with sick building syndrome.

**Conclusion:**

The high prevalence of sick building syndrome symptoms in the nursing environment was associated with factors of unpleasant odor and high workload of environment. So improvement of environmental conditions such as increasing the efficiency of the HVAC system, increasing fresh air flow in the sector, reducing the nurses workload as well as enhancing the quality of working life, will motivate the nurses and increase productivity in the workplace.

## 1. Introduction

Sick building syndrome (SBS) is an indoor air quality related disease, which stimulates the nervous system, skin, and respiratory system, and is accompanied by headaches, dizziness, nausea, coughing, sneezing, irritated mucous membranes of eyes, throat and nose and also inflammation and itching of skin (Hodgson MJ, 2005; Samet, Spengler, & Mitchell, 1998). In this acute disease more than ninety percent of the people affected spend their time in enclosed spaces. Urbanization and limited land for the construction of high-rise buildings has influenced working conditions and has led to the building of structures with inadequate air ventilation. In some cases this can cause SBS in people. These symptoms can be present in office buildings, universities, schools and hospitals (Fischman, 1997; Rollins, 2004). Research has shown that there is an existing relationship between health and the physical environment in which people live, work or are being treated in (Gesler, Bell, Curtis, Hubbard, & Francis, 2004; Ulrich, Quan, Zimring, Joseph, & Choudhary, 2004).

Today, a comprehensive assessment of a building needs qualitative criteria such as thermal comfort in the workplace or residential space which is directly and indirectly linked to satisfaction and comfort of employees and residents of the building (Dinç, 2009; Ganguly, Yunus, Khan, & Malik, 1995; Suh-Hwa Maa et al., 2008). Studies regarding the incidence of SBS in hospitals indicated that the hospital staff, particularly nurses, is under the influence of this syndrome. Moreover, due to their constant presence in the intensive care unit, nurses are more susceptible to harm and disease risks. It is estimated that more than 20% of cases of nosocomial infections occur in ICU patients, deaths occurring as a result of nosocomial infections in the ICU vary from a range of 10 to 80% (Alberti et al., 2002). Epidemiological studies have shown that SBS, and high populations of microbes within indoor airflow are associated (Zhang, 2004). In this context, improving employees’ work-life environment is the concern of many organizations. Quality of work life is a comprehensive program dedicated to promotion and employee satisfaction and therefore also necessary for attracting and maintaining the required staff as well as motivating and bringing out a positive attitude amongst employees (Carr & kazanowski, 1994).

SBS manifests as weakness and fatigue, reduced productivity and increased absence from work. Considering the nurse’s important role in saving the lives of patients and hospital infection control, this is notable. SBS is also a subject to many factors that influence such as contact, exposure to biological factors and chemical pollutants from medical drugs and detergents, disinfectants, detergents, solvents and workplace physical factors such as noise, temperature, humidity and inadequate light and ventilation. The purpose of this study was to evaluate the prevalence of SBS and associated factors among nurses in teaching hospitals of Shahid Sadoughi University of Medical Sciences, Yazd, Iran.

## 2. Materials and Methods

This is a cross-sectional study in which MM040EA questionnaire for sick building syndrome and indoor air quality are evaluated. The questionnaire was translated from English to Persian, and to evaluate its validity, in a pilot study, 10 nurses completed the questionnaire in Yazd and its reliability was confirmed by Cronbach’s alpha test. Shahid Sadoughi University of Medical Sciences has three teaching hospitals. Shahid Sadoughi hospital has an area of 43,000 square meters and is over 10 years old with four floors and central air conditioning system of which its facilities is located outside of the hospital building. Shahid Rahnamoon Hospital is over 35-year old, with two floors and air conditioning system, Afshar Hospital with a total of 22000 meters squared has central air conditioners and exhaust fan. All subjects were asked for consent in order to participate in the study. Subjects were asked using census method; they were then given a questionnaire to answer. The information provided in the questionnaires was analyzed by SPSS software and by chi-square and t tests. Demographic data of the subjects were matched for age and sex, experience, hours work in a week, time shift, the number of daily visitors, Landscape design and building divided, into different groups and were compared. The questionnaire intended to identify those patients symptoms, answer were divided to; yes often, yes sometimes, no never has been, yet the question remains that are these symptoms due to work and the working environment? People who are at least one neurological symptoms like feeling of heaviness in the head, including headaches, nausea, dizziness and difficulty concentrating and a positive sign in the stimulation of the respiratory mucosa irritation and itchy, watery nose, sneezing, dry throat cough, itchy or watery eyes, dry skin and redness, scaling and itching of the scalp, ear, skin redness and dryness from the working environment are considered as positive for SBS (Kubo et al., 2006; Saijo et al., 2009).

## 3. Results

In this study, 265 nurses of Surgical and intensive care units in teaching hospitals of Shahid Sadoughi University of Medical Sciences, participated, which included 42 males (15.8%) and 223 females (84.2%). Their average age was 34.26±7.05. Based on data from the questionnaires, the average total work experience of nurses was 10.22±6.88 years and 70.6 percent of the subjects had worked for less than ten years in the intensive care and surgical section. Prevalence of SBS in the hospital nurses was 86.4% (229 people). SBS prevalence in women, was87% (194 patients) and in men, it was 83.3% (n=35). The difference was not statistically significant between male and female subjects (P=0.52). SBS prevalence in Rahnemoon hospital was (86.7%) and in Sadoughi hospital it was (90.7%) and Afshar Hospital (82.4%), in which a statistically significant association was found between hospital type and prevalence of SBS (P=0.308). In this study Surgical and intensive care unit were included. Based on the prevalence of symptoms of SBS in nurses of ICU (86.1%) 68 personal, CCU 54 patients (87.1%) and NICU 19 patients (82.6%) and Surgery 88 patients (87.1%) had SBS respectively however there was no significant relationship between the department and SBS (P=0.948).

Nurses were divided into 3 groups based on hours working per week; 48 hours, 60 hours and more than 60 hours per week. The results showed that 92.5% of nurses who work more than 60 hours per week have signs of SBS and are affected, this was significant. Association between weekly working hours and SBS was not observed (P=0.34).

Headache (83.3%) and fatigue (89.6%) were the most common general symptoms of SBS and among the irritable respiratory mucosa and skin symptoms, cough with 53.6% dryness of hand (64.9 %) and burning eyes with 50.6% were more common than other symptoms (Table 1).

**Table 1:** Distribution of SBS symptoms in nurses

SBS Symptoms	N	%
Fatigue	238	89.6%
Heavy headed	204	77%
Headache	222	83.3%
Nausea	146	55.1%
Centralization	186	70.2%
Burning eyes	134	50.6%
Runny nose	103	38.9%
Dry throat	103	38.9%
Cough	142	53.6%
Skin redness	126	47.5%
Itching scalp and ears	122	46%
Dry skin	172	64.9%

The operating environmental and odor of the workplace were significantly associated with the prevalence of SBS (P=0.004). So that 90.7% of the nurses who always sense a foul odor and 80.7% of nurses who occasionally sense the fouling odor both show the symptoms of SBS (Table 2).

**Table 2:** Frequency distribution of SBS prevalence by feeling unpleasant odor in the workplace

SBS feeling unpleasant odor	Yes (%)	No (%)	Sum (%)
Yes often	156 (%90.7)	16 (%9.3)	172 (%100)
Yes some times	67 (%80.7)	16 (%19.3)	83 (%100)
No never	6 (%60)	4 (%40)	10 (%100)
Total (P=0.004)	229 (%86.4)	36 (%13.6)	265 (%100)

There was a significant association between lack of the sense of airflow in the working environment with SBS symptoms (P=0.015) and (87.9%) of nurses who work in heavy air often due to poor exhaust air and ventilation complained of symptoms of SBS and (88.4%) of those who sometimes complained about it sometimes show symptoms of SBS (Table 3).

**Table 3:** Frequency distribution SBS prevalence by feeling lack of Airflow

SBS feeling lack of airflow	Yes (%)	No (%)	Sum (%)
Yes often	102 (%87.9)	14 (%12.1)	116 (%100)
Yes some times	114 (%88.4)	15 (%11.6)	129 (%100)
No never	13 (%65)	7 (%35)	20 (%100)
Total (P=0.015)	229 (%86.4)	36 (%13.6)	265 (%100)

In this study, the relation of the prevalence of SBS and other environmental factors such as weather, blizzard (P =0.572), high-temperature environments (P=0.297), contact with static electricity (P =0.11) Cypress sound (P =0.393) and dust (P=0.106) was not statistically significant. But the prevalence of SBS was in relation with the high workload of the nurses (p<0.0001). The data obtained indicated that 95.5% of the nurses that often and % 88.5 of nurses who sometimes complained were suffering SBS (Table 4).

**Table 4:** Frequency distribution of SBS prevalence by workload

SBS High Workload	Yes (%)	No (%)	Sum (%)
Yes often	64 (%95.5)	3 (%4.5)	67 (%100)
Yes some times	131 (%88.5)	17 (%11.5)	148 (%100)
`No seldom	29 (%70.7)	12 (%29.3)	41 (%100)
No never	5 (%55.6)	4 (%44.4)	9 (%100)
Total (P>0.0001)	229 (%86.4)	36 (%13.6)	265 (%100)

## 4. Discussion

Prevalence of SBS among nurses who were studied was 86.4% (229 people) and initially the symptoms of SBS including headache (83.3%) and fatigue (89.6%) were the most common signs of SBS and the symptoms such as respiratory mucosa and skin irritation, coughing (53.6%) dry skin (64.9%) and burning eyes with (50.6%), were more prevalent than other symptoms. Environmental factors at work such as sense of lack of air conditioning, foul odor (P<0.05) were associated with SBS symptoms. From the nurses point of view working conditions have a significant association with SBS (p<0.0001). So that 95.5% of the nurses that often and 88.5% of the nurses who sometimes knew their work load to be increased had the symptoms of SBS. In Sadaghnigat et al studies it was reported that the prevalence of SBS in office buildings was as much as 58.7%. The most common prevalence of symptoms were fatigue (57.3%) and headache (41.5%) and from environmental factors, lack of the sense of air flow (68.4%) and low air quality which promotes choking (59.1%) with (P<0.05) were associated with the prevalence of SBS. On the matter of contributing factors to SBS, the current study findings are consistent with previous studies (Sadeghneeat et al., 2002). Wilson et al. In a study of 4373 employees working in 46 buildings investigated the prevalence of SBS. Based on the results of this study, the prevalence of SBS was eighty percent. The participating subjects’ symptoms included lack of concentration (57%), nasal congestion (47%), dry throat (46%) and headache (43%), respectively. Accordingly, the present study is consistent with the prevalence of SBS that was mentioned (Burge, Hedge, Wilson, Bass, & ROBERTSON, 1987). The results of this study compared with studies conducted in administration and office workplaces shows the high prevalence of SBS can be caused by the peoples active environment and other environmental factors affecting SBS, on a count of this present study being carried out in hospitals. Epidemiologic research has shown that the symptoms of SBS is related to high microbial indoor air (Zhang, 2004). In general, factors such as the low quality of crowded hospital sectors and so many patients in all wards, high humid weather which is conducive to the growth of microorganisms on surfaces and also visiting people, are such factors responsible for the increased of pollution in these areas, contributing to the high prevalence of SBS in the hospitals (Burge HA, 1990; Obbard, & Fang, 2003) In this study, an association was not found between the prevalence of SBS, sex, age, work experience and work shift. However, some studies reported the prevalence of SBS was more in women than men (Nordström, Norbäck, & Akselsson, 1995) and in some cases, the SBS prevalence is not different in both sexes (Brasche, Bullinger, Morfeld, Gebhardt, & Bischof, 2001). In this study, the Prevalence of SBS was in relation to nurses workload p <0.0001, and was obtained such that 95.5% of nurses which in most often, and 88.5% of the nurses that sometimes had a workload increases experienced the known symptoms. In The study performed by Nordström, dissatisfaction with working conditions and the nurses workload were associated with SBS (Nordström et al., 1995). Watzky has shown that intensive care and high working pressure are the main stressor factors that affect nurses in intensive care hospitals in Canada (Sawatzky, 1996). Abdi, in a study conducted on intensive care nurses also reviewed the most important sources of stress to be the physical environment and responsibility (Abdi & Shahbazi, 2002). Previous research has shown that the nursing work environment and physical factors associated with the disease are associated, which is consistent with results of this research.

In a similar study on the impact of indoor air quality, SBS and related factors in an elderly Swedish Hospital, the most common symptoms observed were headaches, fatigue, eye irritation and dryness of the skin. The contributing environmental factors of this study such as exposure to environmental factors affecting static and noise from the ventilation system, symptoms such as headache and fatigue, were found to have similar results as our study (Abdi & Shahbazi, 2002). Research results on the prevalence of SBS in 2011 in Marques University Hospitals of Spain showed that most symptoms and environmental factors declared by staff were such as unpleasant odor, irritation and watery eyes, sore throat, redness and inflammation of the skin as the most common symptoms. Furthermore, no significant relationship was found between volatile organic compounds (VOCS) and microbiological contaminants and SBS (Gómez-Acebo et al., 2011).

The present results declare that 90.7% of nurses with SBS symptoms had often sensed a foul odor during the week at the workplace. This foul odor included toilets, the smell of sewage and septic waste and the smell of medicines, hospital kitchen appliances, cooking odors and chlorine which are the major causes of chemical disinfectant smell in the hospital sector. Also, exposure to the other Physical factors of the work environment such as changes in ambient temperature during the day, the unpleasant smell of air and little fresh air, and sterile work environment, a full exhaust of polluted air due to system defectiveness or lack of capacity exhaust fan and exhaust air are effective on SBS symptoms.

## 5. Conclusions

The high prevalence of SBS in nurses is associated with the work environment unpleasant smell and high workload. Therefore, more attention to the improvement of environmental conditions, such as improved air conditioning system and increasing its efficiency and reducing maintenance time with unpleasant smell and fresh air inlet can be effective. Also reducing the workload of nurses and increasing their awareness of the factors affecting SBS and increasing productivity and enhancing the quality of work life and job satisfaction of nurses working to establish incentive. Therefore all aspects of patient care is evident to SBS studies and wider environment.
